# Pentraxin 3 is a stromally-derived biomarker for detection of pancreatic ductal adenocarcinoma

**DOI:** 10.1038/s41698-021-00192-1

**Published:** 2021-06-29

**Authors:** Michelle R. Goulart, Jennifer Watt, Imran Siddiqui, Rita T. Lawlor, Ahmet Imrali, Christine Hughes, Amina Saad, Joanne ChinAleong, Chris Hurt, Catrin Cox, Roberto Salvia, Alberto Mantovani, Tatjana Crnogorac-Jurcevic, Somnath Mukherjee, Aldo Scarpa, Paola Allavena, Hemant M. Kocher

**Affiliations:** 1 Centre for Tumour Biology, Barts Cancer Institute – a CRUK Centre of Excellence, Queen Mary University of London, London, UK; 2 Barts and the London HPB Centre, The Royal London Hospital, Barts Health NHS Trust, London, UK; 3 Humanitas Clinical and Research Center – IRCCS, via Manzoni 56, Rozzano, Italy; 4 ARC-NET Research Center for Applied Research on Cancer, and Department of Diagnostics and Public Health, Section of Pathology, University of Verona, Verona, Italy; 5 Barts Pancreas Tissue Bank, Barts Cancer Institute- a CRUK Centre of Excellence, Queen Mary University London, London, UK; 6 Centre for Trials Research, Cardiff University, Cardiff, UK; 7 The Pancreas Institute and Department of Surgery, University and Hospital Trust of Verona, Verona, Italy; 8 The William Harvey Research Institute, Queen Mary University of London, Charterhouse Square, London, UK; 9 Centre for Cancer Biomarkers and Biotherapeutics, Barts Cancer Institute – a CRUK Centre of Excellence, Queen Mary University of London, London, UK; 10 Oxford Institute for Radiation Oncology, Churchill Hospital – Oxford Cancer Centre, University of Oxford, Oxford, UK; 11 Present Address: Department of Oncology UNIL CHUV, University of Lausanne, Epalinges, Switzerland

**Keywords:** Tumour biomarkers, Diagnostic markers, Cancer microenvironment

## Abstract

Pancreatic ductal adenocarcinoma (PDAC), characterized by dense desmoplastic stroma laid down by pancreatic stellate cells (PSC), has no reliable diagnostic biomarkers for timely detection. A multi-center cohort of PDAC patients and controls (chronic pancreatitis, intra-ductal papillary neoplasms, gallstones and otherwise healthy) donated serum in an ethically approved manner. Serum PTX3 above 4.34 ng/mL has a higher sensitivity (86%, 95% confidence interval (CI): 65–97%) and specificity (86%, 95% CI: 79–91%), positive predictive value (97%) and likelihood ratio (6.05), and is superior when compared to serum CA19-9 and CEA for detection of PDAC. In vitro and ex vivo analyses of PTX3, in human PDAC samples, PSCs, cell lines and transgenic mouse model for PDAC, suggest that PTX3 originates from stromal cells, mainly PSC. In activated PSC, PTX3 secretion could be downregulated by rendering PSC quiescent using all-trans-retinoic acid (ATRA). PTX3 organizes hyaluronan in conjunction with tumor necrosis factor-stimulated gene 6 (TSG-6) and facilitates stellate and cancer cell invasion. In SCALOP clinical trial (ISRCTN96169987) testing chemo-radiotherapy without stromal targeting, PTX3 had no prognostic or predictive role. However, in STARPAC clinical trial (NCT03307148), stromal modulation by ATRA even at first dose is accompanied with serum PTX3 response in patients who later go on to demonstrate disease control but not those in whom the disease progresses. PTX3 is a putative stromally-derived biomarker for PDAC which warrants further testing in prospective, larger, multi-center cohorts and within clinical trials targeting stroma.

## Introduction

Delayed diagnosis results in up to 90% of patients with pancreatic ductal adenocarcinoma (PDAC) presenting with advanced disease^1^, largely due to the lack of explicit symptoms and signs early in the illness which is exacerbated by the dearth of specific biomarkers to aid early detection^2^. The currently used serum biomarker for PDAC in clinical practice: CA19-9, has a medium range sensitivity (41–86%) and specificity (33–100%)^2,3^. It is suggested that combination of CEA and CA19-9 may increase diagnostic accuracy^4^; however, this has not been replicated^3^. Thus, there are currently no reliable and clinically applicable biomarkers for early detection of PDAC, although a panel of urinary biomarkers is making in-roads into large-scale clinical trial^5^.

PDAC is characterized by an intense desmoplastic stroma, laid down by the pancreatic stellate cells (PSC)^6–8^. Previous work from our laboratory has shown that by targeting the activated PSC with the pleiotropic agent All-trans Retinoic Acid (ATRA) renders them quiescent^9^. Gene-expression microarray revealed that rendering PSC quiescent results in down-regulation of *pentraxin 3 (PTX3*) transcripts. We postulated that serum PTX3 could be a surrogate marker of desmoplasia and, therefore, PDAC.

**Fig. 1:**
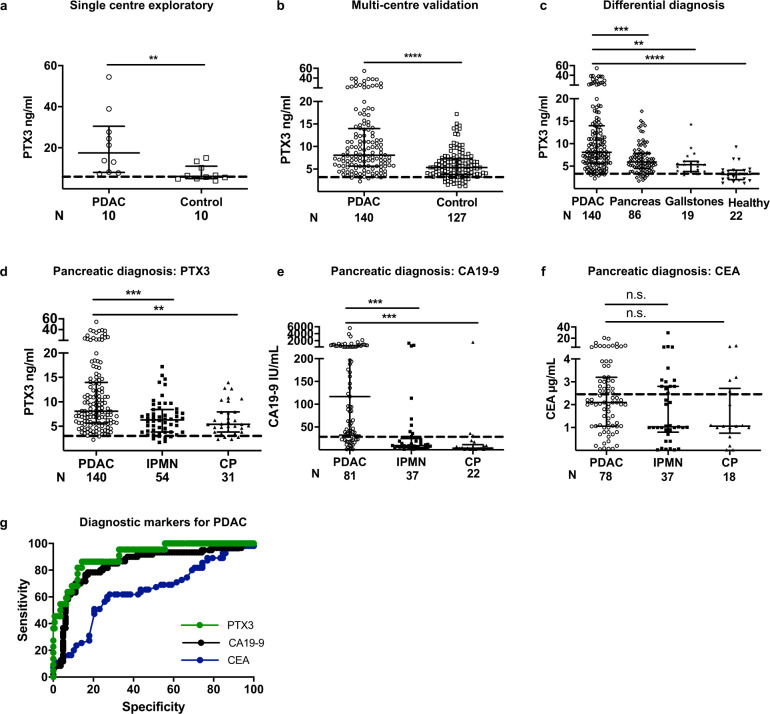
Serum PTX3 is an early detection protein for PDAC. **a** Exploratory single center and **b** validation multi-center cohorts comparing serum PTX3 levels in patients with PDAC and those with non-neoplastic pancreatico-biliary disease and healthy individuals (control). **c** Comparison of serum PTX3 levels between patients with PDAC and those with other pancreatic diseases, gallstones and healthy people. Comparison of **d** serum PTX3, **e** CA19-9 and **f** CEA levels between patients with PDAC and those with IPMN or chronic pancreatitis. **g** Receiver operating characteristic (ROC) curve for PTX3, CA19-9 and CEA. The AUC for PTX3 is 91%, with a cut-off value of 4.35 ng/mL, sensitivity for pancreatic cancer is 86%, specificity 86%, and positive predictive value 97.5%. PTX3 ROC curve was generated by comparing PDAC vs. healthy while CA19.9 and CEA curves were generated by comparing PDAC vs. non-malignant pancreatico-biliary conditions (as data from healthy individuals was not available). Serum PTX3, CEA and CA19-9 levels from healthy volunteers (historical data) are depicted with dotted line. **a**–**f** Summary data are expressed as median and interquartile range. Each data-point is a patient, derived from a median value of three to four PTX3 replicates, but single CA19-9 and CEA measurement. **a**, **b** Mann–Whitney *U* test; **c**–**f** Kruskal–Wallis test and Dunn’s multiple comparisons test; **P < 0.01, ***P < 0.001, ****P < 0.0001.

A pilot study assessing serum PTX3 levels in patients with PDAC along with age-/gender-matched controls (Fig. 1A) and nomograms for diagnostic tests^10^, determined a sample size of 260. This was based on an assumption of 50% prevalence (P~0.5) in our cohort (cancer versus other pancreatic diseases or normal controls), an anticipated accuracy (W~0.05) and a confidence interval of 5% (CI = 0.05, *z* = 1.96) with a sensitivity of 90% (SN = 0.9), and specificity of 90% (SP = 0.9).^11,12^.

In a multi-center cohort (London 101, Verona 157, Milan 23, total 267) including 140 patients with PDAC, we carried out diagnosis- and clinical-characteristics-blinded serum PTX3 measurements, from samples donated at the time of diagnosis (treatment-naïve), using a validated ELISA^13^. Serum PTX3 levels were significantly higher for patients with PDAC than in all controls pooled together (Fig. 1B) or separated as those with other non-malignant conditions of the pancreas (*n* = 86), patients with gallstone disease (*n* = 19) and normal healthy volunteers (*n* = 22) (Fig. 1C). Moreover, patients with PDAC have significantly higher serum PTX3 levels than those with intra-ductal papillary mucinous neoplasm (IPMN) (*n* = 54) or chronic pancreatitis (*n* = 31), common confounders in clinical practice (Fig. 1D). Thus, amongst patients with similar clinical presentation where PDAC is being considered as differential diagnosis, serum PTX3 is better than clinically used tumor-marker CEA and is similar in performance to CA19-9 (Fig. 1E–G, Supplementary Table 1) making a case for a prospective cohort study with matched data-points for formal comparison. Increasing tumor grade, stage, nodal status and presence of metastases did not affect the serum PTX3 levels in those patients with PDAC, although this study was not powered to detect such differences (Supplementary Fig. 1A–D).

As PTX3 is a protein associated with local inflammation^14^, levels of other systemic inflammatory markers (white cell count, CRP), as well as other aspects such as nutrition (albumin) and jaundice (bilirubin), a frequent confounder for raised CA19-9, were also assessed and demonstrated no differences among a sub-set of patients with PDAC and controls, where data was available (Supplementary Fig. 1E–H). These data demonstrate our investigation in a clinically relevant cohort, where differential diagnosis is an ever-present dilemma^1,11^

**Fig. 2:**
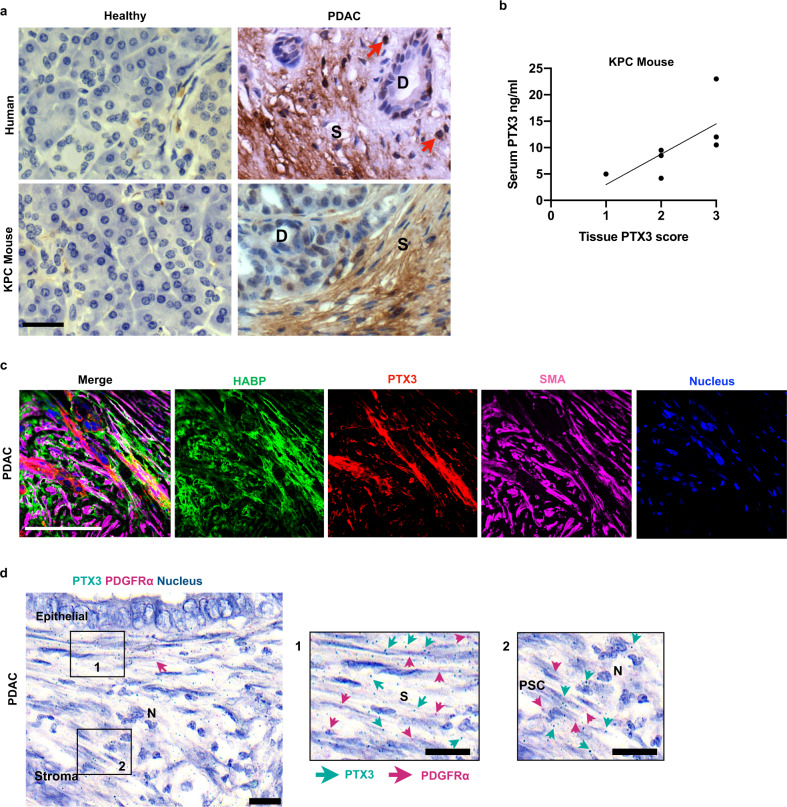
PTX3 is expressed and secreted by pancreatic stellate cells. **a** Representative immunohistochemistry (IHC) images showing expression of PTX3 protein in human (top) and mouse (transgenic KPC and wild-type normal littermate, bottom) pancreatic normal and cancer tissues. Red arrows: neutrophils, D: ducts, S: stroma. **b** Correlation plot between serum PTX3 levels and tissue PTX3 expression in KPC mouse model (*n* = 7). **c** Representative immunofluorescence staining of human PDAC tissue showing co-localization of HABP, PTX3 and α-SMA in the stromal compartment. (Scale bar = 100 µm). **d** RNAscope images of human PDAC demonstrating co-localization of PTX3 transcripts with PDGFα transcripts identifying fibroblasts in the juxta-tumoral space. S: stroma, PSC: pancreatic stellate cells, N: Neutrophil. c: Scale bar = 100 μm. d: Scale bar = 20 μm.

Ex vivo immunostaining for PTX3 in normal human and murine pancreas confirmed that PTX3 was not present in either exocrine or endocrine cells or surrounding stroma. However, PTX3 immunostaining was seen in the stroma of human PDAC, as well as in the most commonly used transgenic mouse model of PDAC, Kras^LSL.G12D/+^, p53^R172H/+^, Pdx^Cretg/+^ (KPC) mouse, with no immunoreactivity in most cancer cells except those within peri-neural invasion (Fig. 2A, Supplementary Fig. 2A–B). PTX3 expression was also seen in endothelial cells and neutrophils (Supplementary Fig. 2C–E), both serving as internal positive controls^12,13,15^.

In a small cohort (*n* = 7), where matched murine samples were available, we demonstrated a strong correlation between serum and tissue PTX3 levels (Fig. 2B). Since the stroma of PDAC is rich in the extra-cellular matrix hyaluronic acid (HA)^16^, and PTX3 binds to tumor necrosis factor alpha-induced protein 6 (TNFAIP6 or TSG6) to stabilize hyaluronan^17^, we next investigated the interaction of PTX3 and HA within the stromal compartment of human PDAC, ex vivo, in human tissue samples. Immunofluorescence microscopy revealed that PTX3 co-localized with HA in the stromal compartment (Fig. 2C). RNAscope of PDAC tissue sections confirmed the abundant PTX3 mRNA expression by the juxta-tumoral PSC (and not cancer cells), which were identified by PDGFRα mRNA expression, with internal positive control being neutrophils identified by nuclear morphology (Fig. 2D), suggesting that tumor cell staining originated from a stromal secreted product. Though epithelial source for PTX3 secretion in other cancer tissues is described^18,19^, our data demonstrate that in PDAC the dominant secretion is from PSC. Further in depth analysis of human samples confirmed the stromal compartment as the main source of PTX3 and HA, the while TSG-6 appeared to be primarily derived from cancer cells (Supplementary Fig. 3, Supplementary Videos 1–3).

**Fig. 3:**
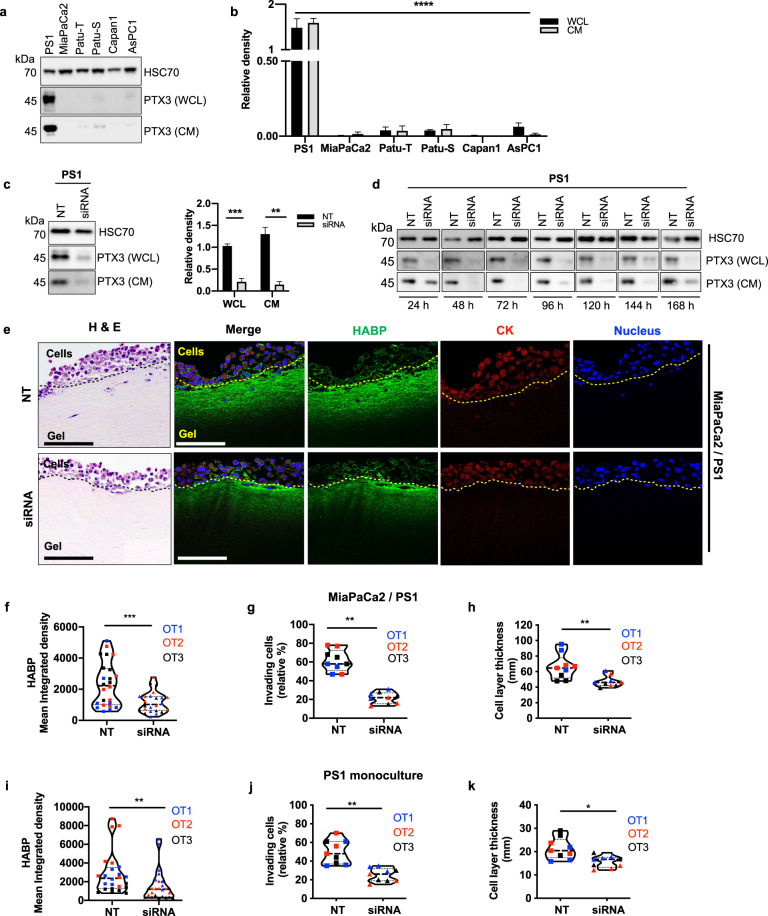
Hyaluronic acid deposition is reduced upon PTX3 siRNA. **a** PTX3 expression in whole cell lysates (WCL) and conditioned media (CM) of pancreatic stellate cell line (PS1), cancer cell lines (MiaPaCa2, Patu-T, Patu-S, Capan1, AsPc1) by Western blot analysis and **b** densitometric quantification (*n* = 4). HSC70 was used as a loading control. **c** Western blot depicting expression of PTX3 in WCL and CM of PS1 cells 72 h after transfection with 20 nM of PTX3 siRNA (siRNA) or non-targeting siRNA (NT), with HSC70 loading control and densitometric quantification (*n* = 3). Summary data are expressed as mean with standard error mean. **d** Duration of PTX3 silencing resulting from siRNA transfection was assessed. Western blot of PTX3 expression in WCL and CM of pancreatic stellate cells (PS1) from 24–168 h. **e** Representative H&E and immunofluorescence images of mini-organotypic cultures of cancer (MiaPaCa2) and stellate (PS1) cells transfected with PTX3 siRNA or NT siRNA, demonstrating HABP expression. Scale bar = 100 μm. Summary quantification data from mini-organotypic co-cultures of cancer (MiaPaCa2) and stellate (PS1) cells transfected with PTX3 siRNA or NT siRNA. **f** Quantification of HABP expression in images (*n* = 6, 8 images/gel). **g** Percentage of invading cells presented as the mean percentage relative to NT control. **h** Cell proliferation assessed by the measurement of the cell layer thickness. Summary quantification data from mini-organotypic monocultures of stellate (PS1) cells transfected with PTX3 siRNA or NT siRNA. **i** Quantification of HABP expression in images (*n* = 6, 8 images/gel). **j** Percentage of invading cells presented as the mean percentage relative to NT control. **k** Cell proliferation assessed by the measurement of the cell layer thickness. Sections from three experimental replicates were analyzed for mini-organotypics, resulting in 27 high-power field measurements (*n* = 9, 3 sections/gel). OT1, OT2, OT3: organotypic biological repeats. **b** Kruskal–Wallis test; WCL, CM: ****P < 0.0001, **c** Mann–Whitney U test, **P < 0.01,***P < 0.001, **f**–**k** Wilcoxon matched-pairs test, *P < 0.05, **P < 0.01, ***P < 0.001. Summary data are expressed as median and interquartile range.

In vitro screening of a panel of pancreatic cancer cell lines and immortalized pancreatic stellate cells (PS1) showed the PTX3 monomer (45 kDa) to be expressed and secreted predominantly by PSC (Fig. 3A–B) and the antibody specificity was confirmed upon PTX3 RNAi (Fig. 3C). At a sub-cellular level, PTX3 staining was localized around the nucleus of PSCs (Supplementary Fig. 4A). In fact, while we were able to demonstrate strong expression of HA in the cytoplasm and the stroma around the PSC, we could not ascertain secretion of TSG6 by PSC (Supplementary Figure 4A). Conversely, cancer cells showed minimal PTX3 and HA expression and strong TSG-6 staining in the cytoplasm (Supplementary Fig. 4B). The expression of PTX3 and HA, but not TSG6 were also confirmed in primary PSCs of all sub-types (A-D) at variable levels, as recently classified by us^20^ (Supplementary Fig. 4C–E). This suggests a symbiotic role of cancer cells and PSC in laying down a therapeutically relevant ECM components such as hyaluronan^16^.

In order to investigate the functional relevance of the interaction of PTX3 with HA in the PDAC stroma, we silenced PTX3 expression in PSC (Fig. 3D, Supplementary Table 2) and introduced them in mini-organotypic co-cultures^21^ (Supplementary Fig. 5A). A rich HA deposition and expression in the ECM gel of mini-organotypic cultures containing PSC alone, by constituting single cell cultures (PS1) as well as co-cultures with cancer cell lines (MiaPaCa2 or AsPC1) was consistently observed, confirming PSC as the source of HA. Furthermore, HA deposition was markedly decreased by silencing PTX3 in PSC, even though the number of PSC remained constant (Fig. 3E–K, Supplementary Figs 5, 6). Intriguingly, we found that silencing PTX3 significantly suppressed the invasion capacity of stellate and cancer cells, in both co-cultures and monoculture experiments (Fig. 3G–K, Supplementary Figs 5, 6). These results support that PTX3 expression by activated PSC is required for HA deposition in the ECM stroma, and is required to facilitate PSC invasion and consequently cancer cell invasion.

**Fig. 4:**
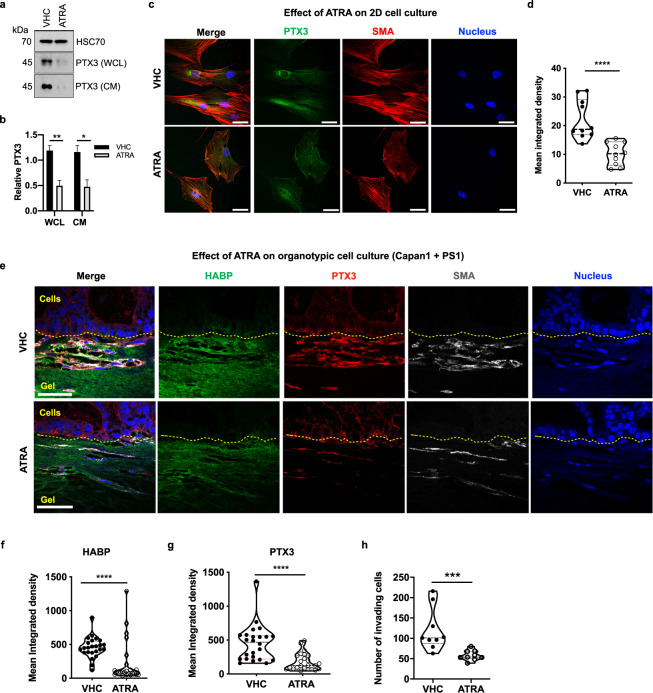
Retinoic acid treatment reduces the expression of the PTX3 protein in vitro. **a** Representative Western blot of PTX3 expression in WCL and CM of pancreatic stellate cells after ATRA or vehicle (VHC) treatment, with HSC70 (WCL) and Ponceau (CM, not shown) used as loading control and its **b** densitometric quantification (*n* = 4). **c** Representative immunofluorescence images of PS1 cells treated with ATRA or vehicle and **d** quantification of PTX3 expression (*n* = 10). **e** Representative immunofluorescence images of mini-organotypic cultures of Capan1 cells / PS1 treated with ATRA or vehicle and **f** quantification of HABP expression in images (*n* = 6, 8 images/gel). **g** quantification of PTX3 expression in images (*n* = 6, 8 images/gel), and **h** quantification of invading cells (*n* = 6, 3 images/gel). **c**, **e** Scale bar = 50 µm. **b** Unpaired *t-*test, summary data expressed as mean with standard error mean. **d**, **f**, **g**, **h**: Mann–Whitney *U* test, *P < 0.05, **P < 0.01, ****P < 0.0001. Summary data are expressed as median and interquartile range.

**Fig. 5:**
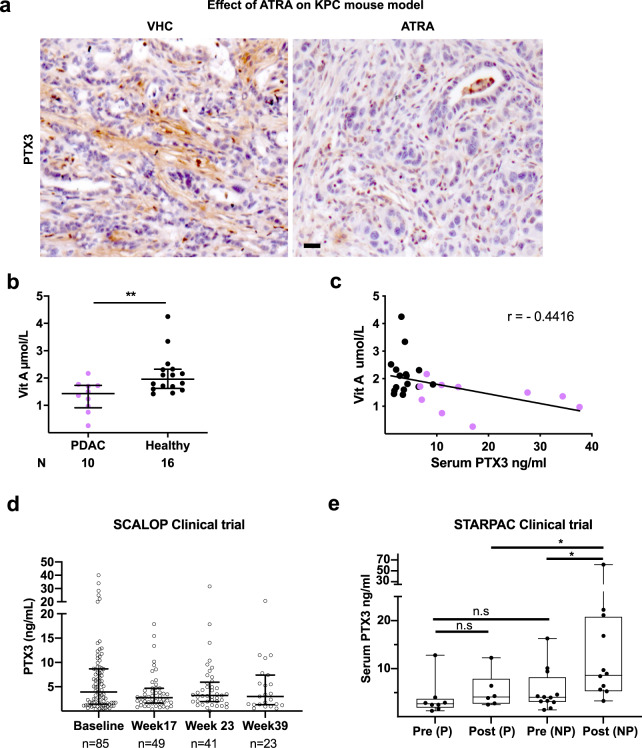
Retinoic acid treatment decreases the expression of the PTX3 protein in mice and humans. **a** Representative immunohistochemistry images of PTX3 expression in pancreatic cancer tissue after ATRA or vehicle treatment in KPC mouse model. Scale bar = 50 μm. **b** Serum vitamin A levels in patients with PDAC (*n* = 10) in comparison with healthy controls (*n* = 16), Mann–Whitney *U* test, **P < 0.01, summary data expressed as median and interquartile range. **c** Correlation plot between serum vitamin A and PTX3 levels in patients with PDAC and healthy subjects. Pearson *r* = −0.44, (95% CI: −0.71 to −0.07), *p* = 0.024). **d** Serum PTX3 levels in a cohort of patients with PDAC from SCALOP clinical trial. **e** Serum PTX3 levels in patients on STARPAC clinical trial as measure before (pre) and 5 h after (post) giving the first ATRA dose (Cycle 1, Day 1). Comparison are made between those patients who progressed (P) and not progressed (NP = stable disease and partial response), Mann–Whitney *U*-test. *P < 0.05, n.s = not significant, summary data expressed as median and interquartile range.

In vitro, in 2D cultures, we demonstrated that ATRA-induced quiescent PSC^9^ express and secrete less PTX3 than their activated counterparts (Fig. 4A–D). Importantly, in 3D organotypic co-cultures, which do not pre-contain hyaluronan^21^, but is deposited by PSC actively, the reduction in PTX3 expression induced by ATRA was also associated with reduced hyaluronan stabilization and invasion (Fig. 4E–H). In fact, we demonstrated a direct co-localization of the activation marker αSMA of the invading PSC in the ECM gel, with the strongest PTX3 and HA expression in contrast to the minimal PTX3 and HA expression seen in the epithelial compartment, strongly suggesting their origin in PSC, contributing to ECM deposition in this reductionist, short-lived, but physio-mimetic co-cultures. Importantly, we also demonstrated down-regulation of PTX3 expression in vivo in the KPC mouse model upon ATRA treatment compared to vehicle treatment^9^ and in primary pancreatic stellate cells (Fig. 5A, Supplementary Fig. 7).

Patients with PDAC demonstrated significantly lower levels of vitamin A in our cohort, (Fig. 5B) as indicated previously by case-control association studies^22^. Of note, in human samples, we demonstrated a correlation between serum PTX3 and vitamin A levels, but no other correlation could be found with any other confounding inflammatory markers (Fig. 5C, Supplementary Table 3).

In the SCALOP clinical trial^23^ (ISRCTN 96169987) that tests the rationale for consolidation chemo-radiotherapy (gemcitabine or capecitabine based radiotherapy) options after induction gemcitabine-capecitabine chemotherapy for 12–16 weeks, where stroma was not specifically targeted, serum PTX3, collected either at baseline (85/114 patient samples available) or post induction chemotherapy (week 17, 49 / 74 patient samples available) or post chemo-radiotherapy (week 23, *n* = 41) or at follow-up (week 39, *n* = 23) was neither predictive nor prognostic for overall survival or progression (Fig. 5D, Supplementary Tables 4–9). There was also no correlation with tumor size, age, gender, treatment arms, CA19-9 levels or inflammatory markers such as absolute neutrophil count (Extended data: Tables 4–9). Furthermore, in a phase I clinical trial targeting stroma with ATRA along with standard chemotherapy^24^ for patients with pancreatic cancer (STARPAC: NCT03307148) we demonstrate that within 5 h after the first dose of ATRA (cycle 1 day 1) there is a significant immediate PTX3 response (increased serum level) in patients with stable disease (non-progressors = stable disease and partial responders) but not in those who eventually have progressive disease (Fig. 5E), with good correlation between serum PTX3 levels and tissue PTX3 expression (Supplementary Fig. 8). This early indicator, albeit with a very small sample size suggests that before starting chemotherapy with ATRA, there is no difference in baseline serum PTX3 levels. The validity of this pharmacodynamic stromal response of PTX3 will need to be determined in larger clinical trial (STARPAC2: NCT04241276).

In summary, serum PTX3 is potentially a sensitive and specific diagnostic biomarker for PDAC with the ability to separate PDAC from other pancreatic diseases such as IPMN and chronic pancreatitis, which pose frequent diagnostic dilemmas in clinical practice (Supplementary Fig. 8). Computerised tomography (CT) scanning is the recognized gold standard imaging in the diagnosis of PDAC. Whilst CT is able to accurately detect the presence of pancreatic masses it is not able to distinguish malignant from benign conditions, where PET-CT may be more useful^25,26^. This is of particular relevance as patients with chronic pancreatitis may mimic PDAC on presentation without overt malignancy, but also have increased risk of malignancy^1^. In light of these diagnostic challenges, PTX3 may be a sensitive and specific biomarker able to distinguish malignant from benign conditions of the pancreas, and merits further exploration in conjunction with other biomarkers to improve its sensitivity and specificity for routine clinical application.

A combination of human tissue and serum samples analyses, and experimental data based on both in in vitro 2D and 3D as well as in vivo transgenic mouse models suggest that the source of elevated serum PTX3 is the desmoplastic stroma of PDAC, in particular the activated pancreatic stellate cell. The short pentraxins such as C-reactive protein (CRP) and Serum Amyloid P component (SAP) are produced in the liver by hepatocytes. In contrast, PTX3, also an acute phase protein, but a long pentraxin, is rapidly produced at the local sites of inflammation by a variety of cell types such as macrophages, neutrophils, endothelial cells, dendritic cells, fibroblasts and smooth muscle cells in response to a myriad of local pro-inflammatory signals such as lipopolysaccharide (LPS), interleukin-1 (IL-1) and tumor necrosis factor-alpha (TNF-α) and Toll-like receptor activation^14,27^. These key signals are highly expressed in the pan-stromal compartment^9,28,29^. We postulate that PTX3 secretion in association with hyaluronan, particularly in the pan-stromal compartment, may entrap immune cells, thus preventing immune cells from accessing tumor cells to mount anti-tumor immunity^28,30^. Whilst PTX3 potentially interacts with a variety of ligands and may be involved in various biological functions, including innate immune responses; here we suggest an essential role for PTX3 in PDAC in stabilizing the ECM: previously understood to be via interaction with TSG-6 and hyaluronic acid^14,17,31^. Furthermore, we demonstrate its role in contributing to the invasive capacity of cancer cells. This leads to the possibility that the stromal modulating treatments such as those using PEGPH20^16^, hedgehog inhibition^32^, or ATRA can be monitored using serum PTX3 levels as surrogate marker of therapeutic response to stromal targeting therapy, since repeated biopsies are not feasible in patients with PDAC^33^.

## Methods

### Human tissue and serum samples

Tissues and serum were collected in an ethically approved manner from patients and volunteers in the UK [Barts Pancreas Tissue Bank, London, UK, https://www.bartspancreastissuebank.org.uk/, Ethical approval by NRES Committee South Central-Hampshire on 23^rd^ January 2014 with reference 11/SC/0592 and renewed as 18/SC/0630}^34^ and Italy [ARC-Net Biobank, Verona, Italy, https://bcnet.iarc.fr/about/partners_arc_net.php, Ethical approval by Integrated University Hospital Trust (AOUI) Ethics Committee (Comitato Etico Azienda Ospedaliera Universitaria Integrata) with Prog. No. 2172 dated 23/05/2012 Prot. N. 26773)^35^. All patients provided written informed consent prior collection of samples. Controls included patients with non-neoplastic pancreatico-biliary diseases as well as healthy individuals. Prospectively collected samples obtained from the UK Medicine and Healthcare products Regulatory Agency approved SCALOP (ISRCTN 96169987) clinical trial^23^ and STARPAC^24^ were analyzed blindly and serum PTX3 assay data sent back to respective clinical trial units for clinical correlates. Serum and tissue samples were obtained from previously conducted experiments on tumor-bearing KPC mice receiving either ATRA or vehicle for two doses over a week and then sacrificed^9,36^.

### ELISA

PTX3 ELISA was performed, in a blinded manner, using in-house validated methodology at Humanitas Research Institute, Milan Italy^12^. Briefly, Plasma PTX3 concentrations were quantified using the sandwich ELISA as follows: 96- well-ELISA plates were coated with MNB4 anti-human PTX3 antibody (100 ng/ well) diluted in coating buffer (15 mM carbonate buffer, pH 9.6, overnight, 4 °C), washed, blocked (5% dry milk in washing buffer, 2 h, room temperature), washed, and incubated with either 50 μL of diluted plasma (1:3 dilution in PBS without Ca^2+^ Mg^2+^ and 2% BSA) or 50 μL recombinant human PTX3 standards (0.31–20 ng/mL), all in duplicates for 2 h at 37 °C. After two washes, 50 ng/mL of biotinylated PTX3 (Enzo Life Sciences, cat. ALX-210-365B) antibody was added in each well for 1 h at RT, washes and color realized by 100 μL/well streptavidinhorseradish peroxidase (Amersham, cat. RPN4401V) diluted 1:4000 for 1 h at RT. After further washes 100 μL of chromogen substrate (ThermoFisher cat. 34028B) was added and plates were read after 15 min at 450 nm in a plate-reader. Polynomial regression graphs were constructed for standard curves.

### Western blotting

Briefly, 20 µg of cell lysates or 20 µL of filtered conditioned media from cell cultures were separated by electrophoresis on 10% sodium dodecyl sulfate/polyacrylamide gels. Membranes were blocked with 5% bovine serum albumin (BSA) in TBST (tris-buffered saline with 0.5% Tween 20), and probed with primary antibodies to PTX3 (Cat. No HM2242, clone MNB4; Hycult Biotech at 1:100 dilution) or HSC70 (Cat. No sc-7298, Santa Cruz at 1:1000 dilution) at 4^o^C overnight followed by appropriate secondary HRP-conjugated antibody (1 h, room temperature) with intervening washes in TBST as described before^37,38^. Immunoreactive proteins were detected with Luminata Forte Western HRP substrate (Millipore). Quantification of independent Western blots (3–4 replicates) was performed using Fiji-ImageJ, (NIH,USA) software. Gels derived from the same experiment were processed in parallel.

### Immunofluorescence

For coverslips, cells were fixed (4% formaldehyde/PBS), permeabilized (0.1% Triton X-100/PBS), blocked in PBST (PBS with 0.1% Tween 20) 2% BSA, and incubated with appropriate primary antibodies (PTX3 (Cat. no. HM2242, clone MNB4; Hycult Biotech at 1:1000 dilution), TSG-6 (Cat. no. PA5-47253, Invitrogen at 1:100 dilution), αSMA (Cat. no. M0851, clone 1A4; Dako at 1:200 dilution), cytokeratin (Cat. No Z0622, Dako at 1:100 dilution) or biotinylated hyaluronan binding protein (HABP, Cat. no. 385911, Merck Millipore at 1:100 dilution), followed by the appropriate secondary antibodies (anti-rat Alexa-488 (Cat. no. A11006), anti-mouse Alexa-633 (Cat. no. A21052), anti-mouse Alexa-546 (Cat. no. A11030), anti-rabbit Alexa-546 (Cat. no. A11035), anti-goat Alexa-488 (Cat. no. A32814), streptavidin Alexa-488, Cat. no. S11223, all Invitrogen at 1:500 dilution), and counterstained with 4′,6-diamidino-2-phenylindole (DAPI) for nuclei visualization (Cat. no. P36931, Invitrogen). Controls with species-specific immunoglobulin were uniformly negative.

For immunofluorescence staining of paraffin sections, 4 µM sections were dewaxed, rehydrated, antigen retrieved (10 mmol/L citrate buffer, pH 6.0, 10 m), blocked (PBST 5% goat serum, 1 h) and incubated with primary antibodies (PTX3 (Cat. no. SAB4502545, Sigma at 1:100 dilution), TSG-6 (Cat. no. PA5-47253, Invitrogen at 1:100 dilution), αSMA (Cat. no. M0851, clone 1A4; Dako at 1:200 dilution), cytokeratin (Cat. no. Z0622, Dako at 1:100 dilution)), E-cadherin (Cat. no. ab1416, clone HECD-1; Abcam at 1:100 dilution), or epCAM (Cat no. MA5-12436 at 1:100 dilution) and HABP (Cat. no. 385911, Merck-Millipore at 1:100 dilution) overnight at 4^o^C with appropriate concurrent negative controls. After washes, the sections were incubated at room temperature with the relevant secondary antibodies (as above) for 1 h at RT. Sections were then mounted onto slides using DAPI for nuclear visualization.

### Immunohistochemistry

Immunohistochemical analysis of human tissues (Super Sensitive IHC kit, (BioGenix) and murine tissues (Vectastain mouse kit, VectorLabs (Burlingame CA, USA)) was carried out as per manufacturer’s instructions. Controls with species-specific immunoglobulin were uniformly negative.

### RNA in situ hybridization (RNAscope)

mRNA in situ hybridization (ISH) was measured with RNAscope 2.5 HD duplex reagent assay (Cat. no 322430 Advanced Cell Diagnostics, ACD, CA) according to the manufacturer’s protocols. Slides were hybridized with Hs-PTX3 probe (ACD, Cat. no. 517611-C1), Hs-PDGFRA probe (ACD, Cat. no. 604481-C2) and negative control probes DapB-C1/ DapB-C2 (ACD, Cat. no. 320751).

### Mini-organotypics

Human PDAC cell lines were obtained from the American Type Culture Collection (ATCC, Rockville, USA). PS1 cell line (immortalized non-tumoural PSC) was developed and characterized previously in our laboratory^39^. The mini-organotypic model^37^, was used to examine the effect of genetic manipulation or ATRA treatment on PDAC cells (MiaPaCa2 and CaPan1) and PSC (PS1) co-cultured in 3D, physio-mimetic environment. Mini-organotypic cultures were incubated for 7 days before being harvested, fixed in 10% neutral buffered formalin (BAF‐0010‐03 A; CellPath Ltd), submerged in 70% ethanol, embedded in paraffin and cut into 4 µm sections. Treatment of organotypic cultures was performed daily with 1 µM ATRA (Cat No. R2625; Sigma) in 100% ethanol or vehicle control. For organotypic cultures using PTX3 siRNA, stellate cells were first transfected with PTX3 siRNA or non-targeting (NT) control, harvested after 24 h and then plated into organotypic gels, and incubated for 5 days. Cancer cell lines (MiaPaCa2 or AsPC1) and stellate cell line (PS1: with NT siRNA or PTX3 siRNA) were used. Medium below the inserts were replaced daily and collected (undernatant) at the end of the cultures for further analysis. Each experiment had three technical replicates with three biological repeats.

### Small interfering RNA

Pancreatic stellate cells were plated in a 6-well plate, transfected with 20 nM of a pool of human PTX3 siRNA (ON-TARGETplus SMART pool, Dharmacon) or with a pool of nontargeting siRNA (ON-TARGETplus non-targeting pool, Dharmacon) in OptiMEM (LifeTechnologies), using INTERFERin transfection reagent (Polyplus Transfection) as per manufacturer’s instructions.

### MTS assay

The MTS (3-(4,5-dimethylthiazol-2-yl)-5-(3-carboxymethoxyphenyl)-2-(4-sulfophenyl)-2H-tetrazolium) cell viability assay was performed according to the manufacturer’s instructions (b197010, Abcam). Cell viability was assessed by measuring OD490 on a plate reader and calculated using according to the following formula: cell viability rate (%) = (OD490 of siRNA cells/OD490 of NT control) × 100%.

### Quantification

The quantification of all cell counts and intensity of staining was performed in at least three representative images per mini-organotypic gel, and each experiment had three technical repeats for each biological replicate. For HABP and PTX3 staining on mini-organotypics, eight representative images per gel were acquired using a confocal laser scanning microscope LSM 880 (Carl Zeiss); the intensities of fluorescence in the green/red channels were assessed in the gel compartment (by ROI) using Fiji-ImageJ software. For quantification of PTX3 staining on coverlips treated with ATRA or vehicle control, five representative images per condition were acquired using a confocal laser scanning microscope LSM 880 (Carl Zeiss) and the intensity of fluorescence in the green channel were assessed using Fiji-ImageJ software. For invasion, cell layer thickness and total cell numbers, siRNA mini-organotypics were scanned using a Pannoramic 250 High Throughput Scanner (3DHISTECH). Representative images of each gel were divided in three sections of the same size, and the number of invading cells were counted in each section. Individual values were normalized and represented as the mean percentage relative to NT control in each experiment. Cell layer thickness were calculated by measuring three different segments in each of section of the gel, while total cell numbers were assessed by counting the cells in each section using Pannoramic Viewer Software (3DHISTECH). The mean of all three analyzed sections were used.

### Statistical analysis

Statistical analysis and graphical data representation were performed using Prism v.8.2 (GraphPad Software, Inc, La Jolla, CA, USA). Summary data are expressed as the median with interquartile range, since the distribution was non‐Gaussian. Unless stated otherwise, non-parametric Mann–Whitney *U* test was used to compare two groups for continuous variables, and one-way ANOVA using Kruskal–Wallis test with Dunn’s multiple comparison tests was performed to compare more than two groups. The level of significance was set at *p* < 0.05.

### Reporting summary

Further information on research design is available in the Nature Research Reporting Summary linked to this article.

## Supplementary information

Supplementary Information

Reporting Summary

Supplementary Video 1

Supplementary Video 2

Supplementary Video 3

## Data Availability

The datasets generated during the current study are available from the corresponding author on reasonable request.
