# Supplementary material for: Pentraxin 3 is a stromally-derived biomarker for detection of pancreatic ductal adenocarcinoma
Source: NPJ Precis Oncol. 2021 Jun 29;5:61. doi: 10.1038/s41698-021-00192-1 (PMC8242009; doi:10.1038/s41698-021-00192-1)
Supplement: Supplementary file 1 — Supplementary Information [file 41698_2021_192_MOESM1_ESM.pdf]

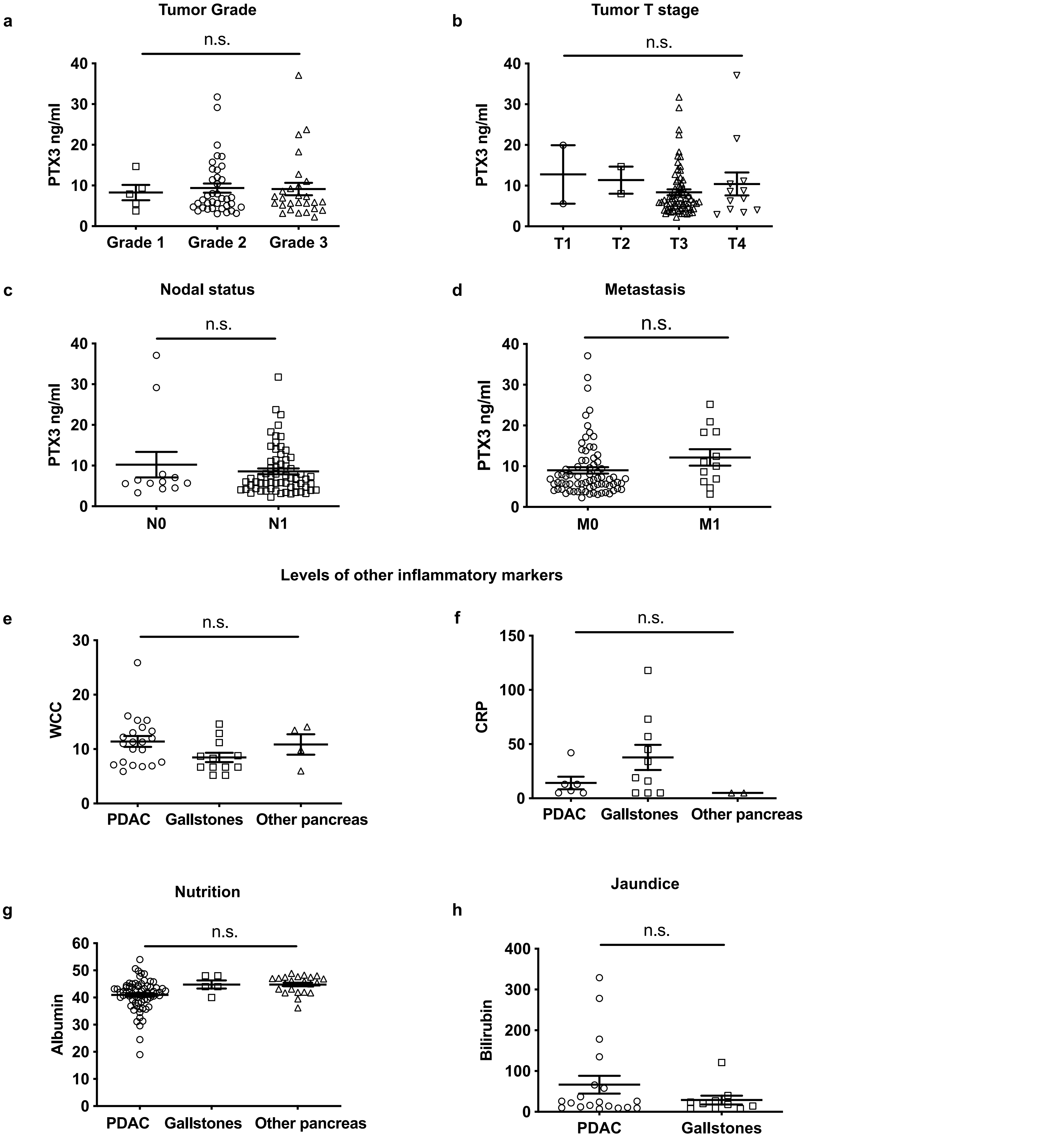

Supplementary Figure 1: Tumor pathological status and confounding factors. Serum PTX3 levels in relation to tumor grade (a), stage (b), nodal status (c), distant metastatic disease (d), white cell count (WCC) (e), serum CRP levels (f), serum albumin levels (g), and serum bilirubin levels (h). a, b, e, f, g: *Kruskal-Wallis* test, c, d, h: *Mann-Whitney U* test, n.s. = not significant. Summary data are expressed as mean with standard error mean.

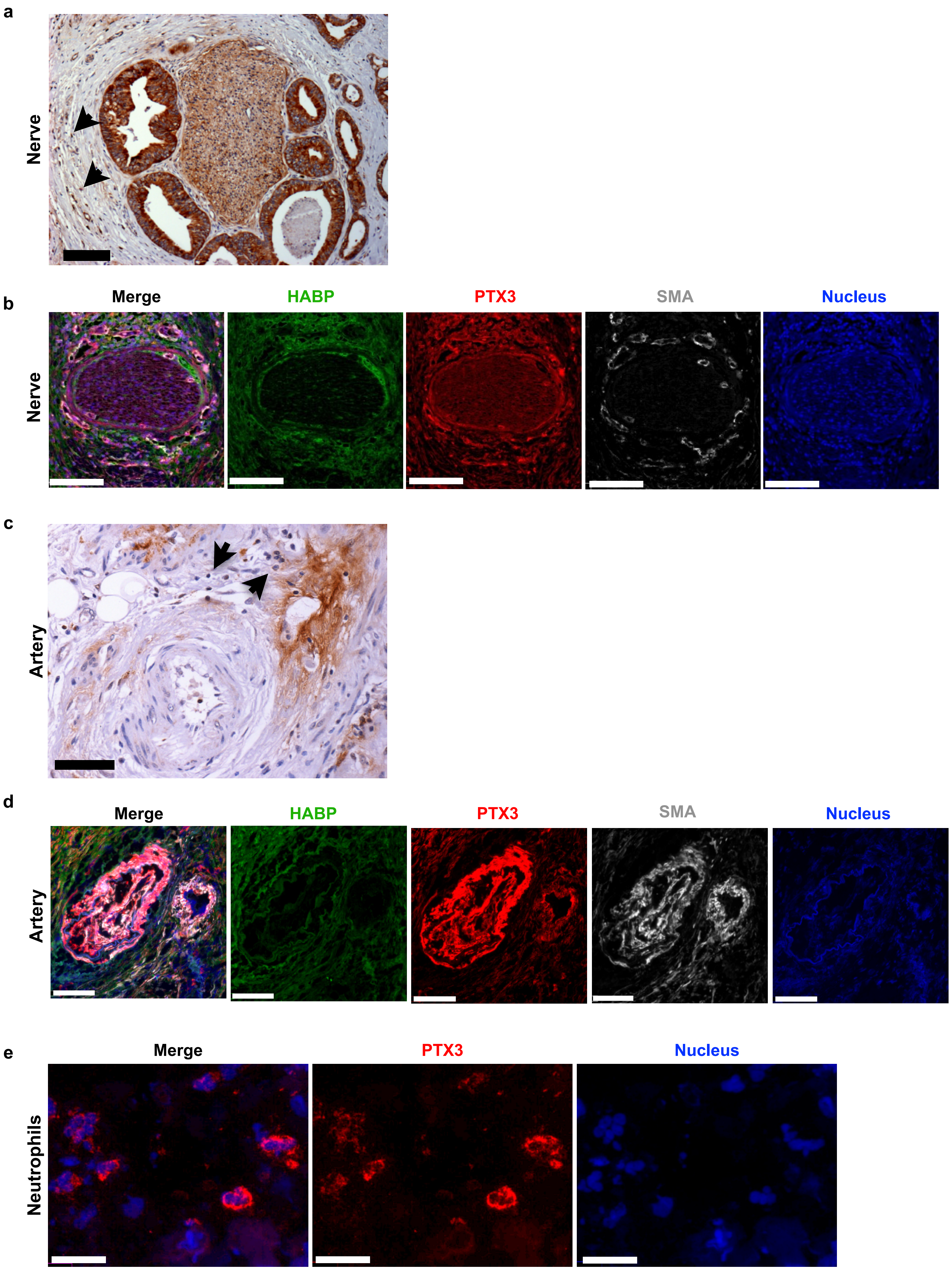

Supplementary Figure 2: PTX3 expression in other tissues. Representative IHC (a-c) and immunofluorescence (b,d,e) images showing PTX3 expression in neural tissues (a, b) peri-vascular areas and endothelial cells (c, d), and neutrophils (e). Arrowheads: neutrophils. a, b, c, d: *Scale bar* = 100µm, E: *Scale bar* =20µm.

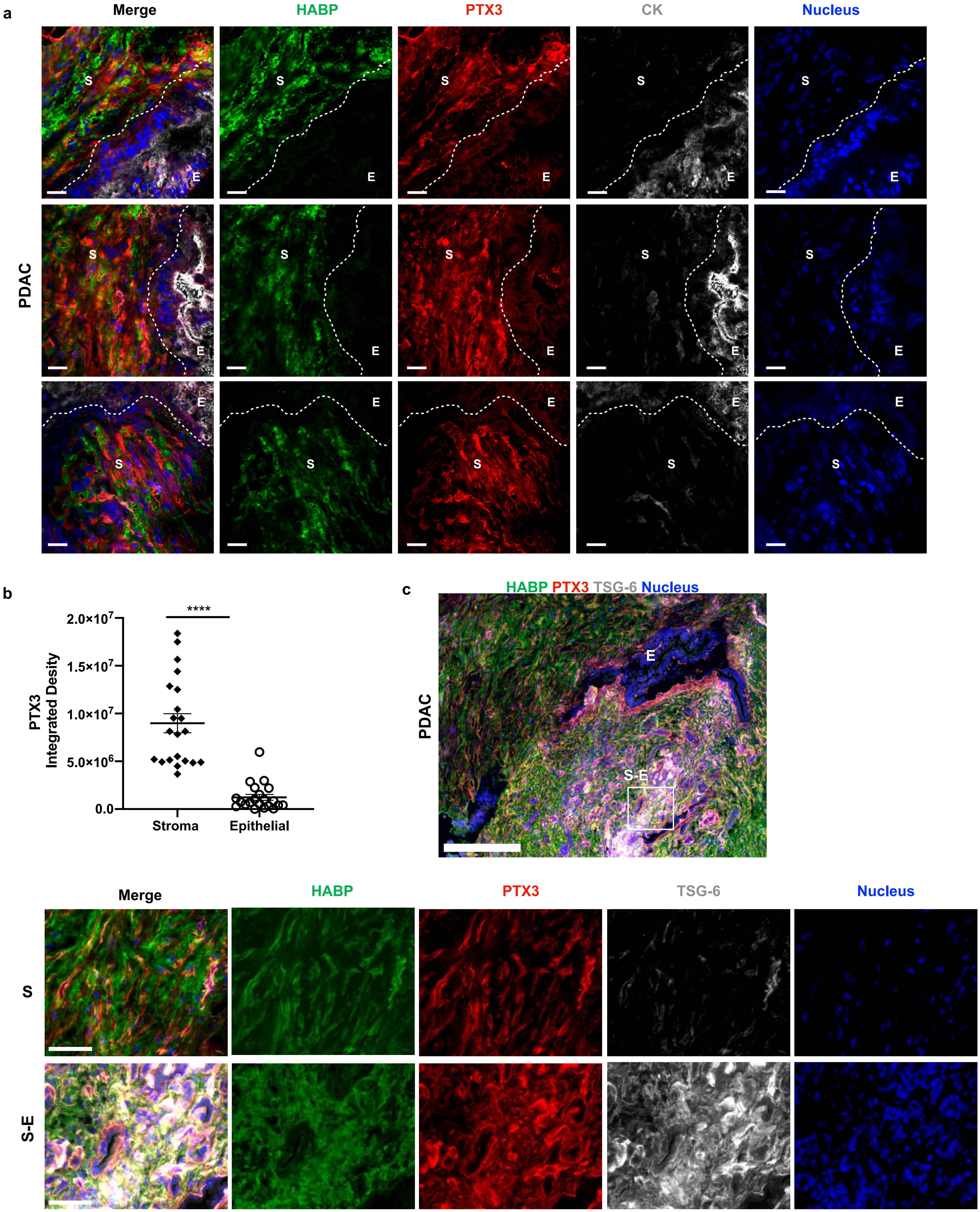

Supplementary Figure 3: HABP, PTX3, CK and TSG-6 distribution in human PDAC. (a) Representative immunofluorescence images showing expression of HABP, PTX3 and CK in human PDAC tissue and (b) Quantification of PTX3 in the stromal and epithelial cell compartment in images (n= 7, 3 area / image). Mann Whitney U test,**** P < 0.0001. Summary data are expressed as mean with standard error mean. (c) Representative immunofluorescence staining showing expression of HABP, PTX3 and TSG-6 in human PDAC tissue with magnification of selected areas. a: *Scale bar* = 100μm c: *Scale bar* = 250μm. S,E: *Scale bar* = 50μm. E: Epithelial, S: stroma. Representative images also shown on supplementary videos 1, 2, and 3.

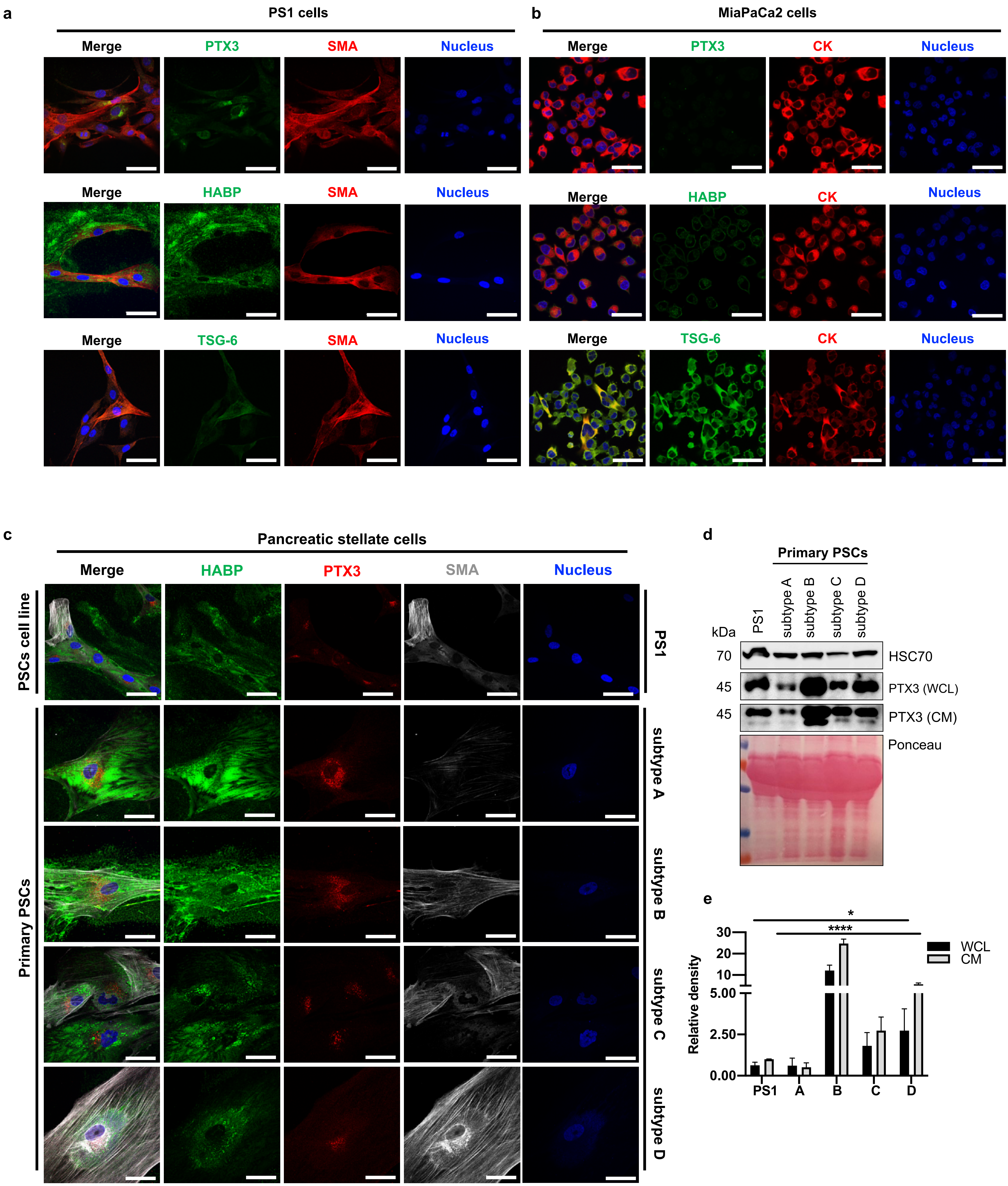

Supplementary Figure 4: PTX3 is expressed and secreted by pancreatic stellate cells. (a-b) Representative immunofluorescence images showing expression of PTX3, HABP and TSG-6 in pancreatic stellate (PS1) cells and cancer (MiaPaCa2) cells. (c) Representative immunofluorescence confocal images demonstrating expression of both HABP and PTX3 in the four different subtypes of primary stellate cells. (d) Western blot of PTX3 expression in whole cell lysates (WCL) and conditioned media (CM) of primary stellate cells and PS1 with associated Ponceau and (e) densitometric quantification (n=3). Kruskal-Wallis test; WCL:*P < 0.1, CM: **** P < 0.0001. Summary data are expressed as mean with standard error mean. *Scale bar* = 50µm.

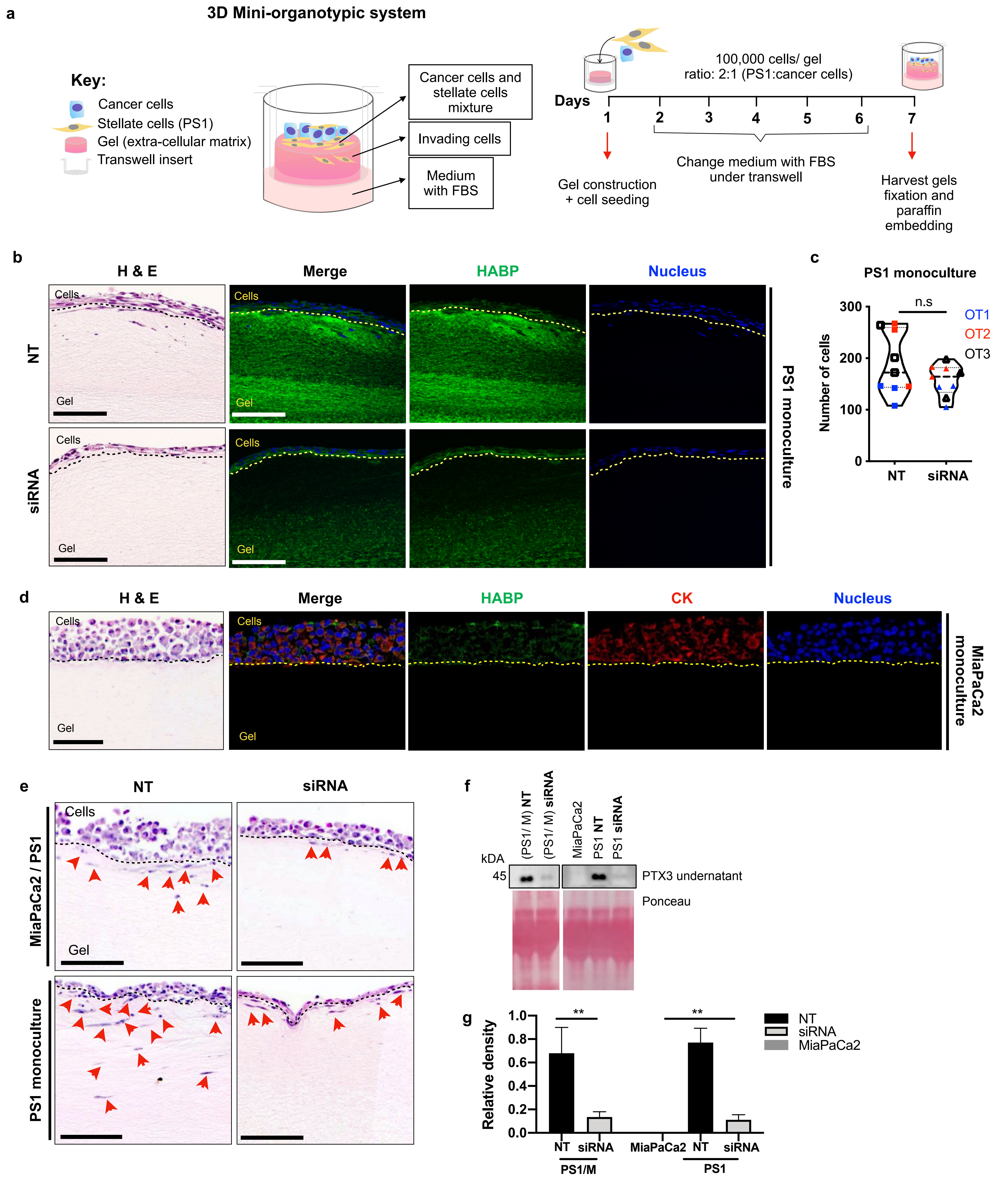

Supplementary Figure 5: PTX3 siRNA knockdown and invasion. (a) Schematic presentation of the 3D mini-organotypic culture model. (b) Representative H&E and immunofluorescence images demonstrating expression of HABP in mini-organotypic monocultures of stellate (PS1) cells, transfected with PTX3 siRNA or NT siRNA. (c) Quantification of cell numbers in PS1 monocultures. Sections from three experimental replicates resulting in 27 high-power field measurements (n = 9, 3 sections/gel). Summary data are expressed as median and interquartile range, Kruskal-Wallis test n.s= not significant. OT1, OT2, OT3: organotypic biological repeats. (d) Representative H&E and immunofluorescence images of mini-organotypic monocultures of cancer (MiaPaCa2) cells. (e) Representative H&E demonstrating cell invasion and proliferation of mini-organotypic cultures with cancer (MiaPaCa2) and stellate (PS1) cells transfected with PTX3 siRNA or NT siRNA. *Scale bar* = 100 μm. Arrowheads: invading cells. (f) Western blot of PTX3 expression from culture medium (undernatant) collected at the end of the mini-organotypic cultures and (g) densitometric quantification (n=3). PS1 (NT), PS1 (siRNA) and MiaPaCa2 monocultures, Kruskal-Wallis test **P < 0.01. PS1/M: Unpaired t test **P < 0.01. Summary data are expressed as mean with standard error mean.

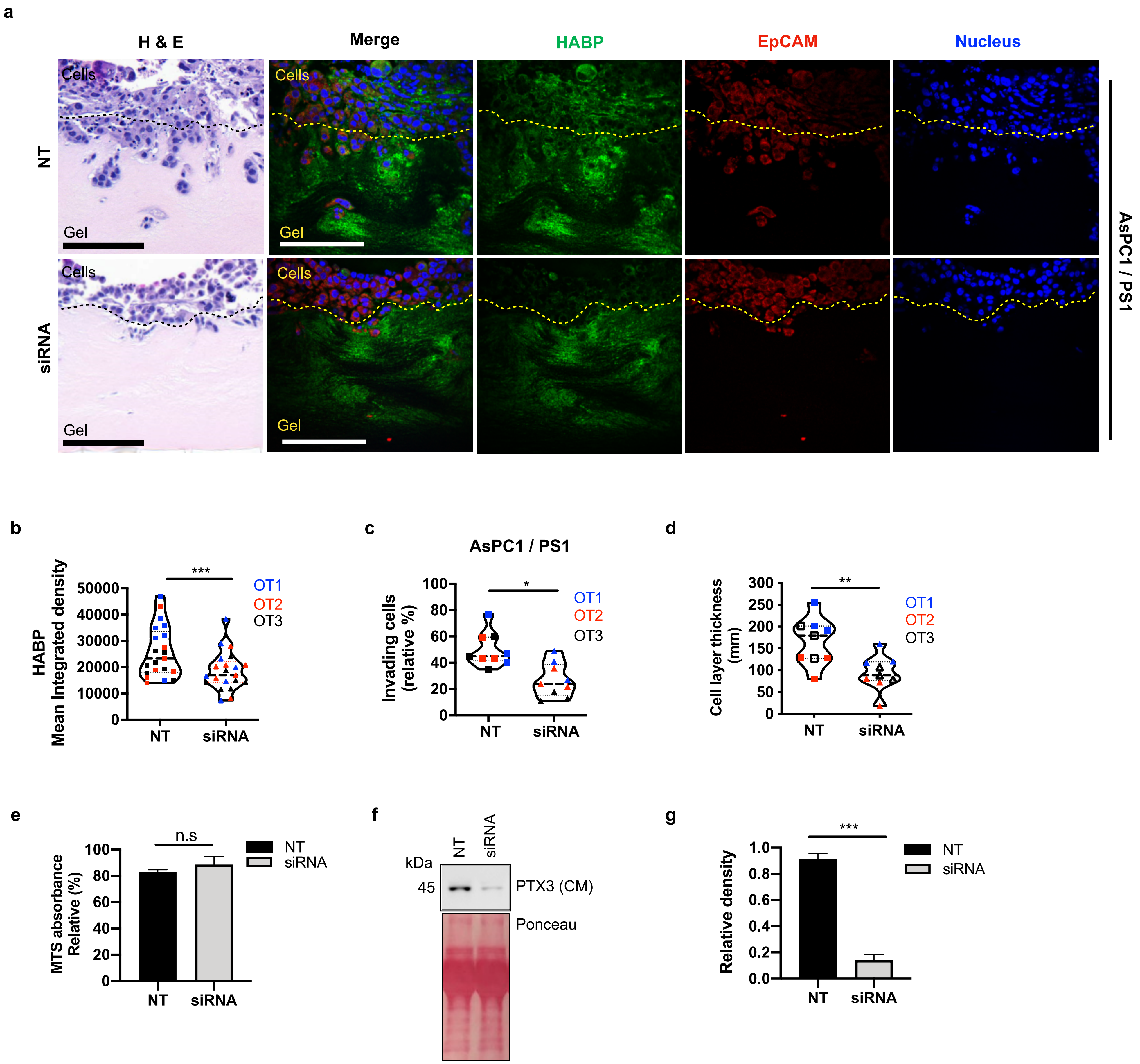

Supplementary Figure 6: Invasion in organotypics after PTX3 siRNA. (a) Representative H&E and immunofluorescence images demonstrating expression of HABP in mini-organotypic cultures with cancer (AsPC1) and stellate (PS1) cells transfected with PTX3 siRNA or NT siRNA and (b) quantification of HABP expression in images (n=6, 4 images/gel). *Scale bar* =100µm (c) Percentage of invading cells presented as the mean percentage relative to NT control. (d) Cell proliferation assessed by the measurement of the cell layer thickness. Sections from three experimental replicates were analyzed for mini-organotypics, resulting in 27 high-power field measurements (n = 9, 3 sections/gel). OT1, OT2, OT3: organotypic biological repeats. Wilcoxon matched-pairs test,* $P < 0.05$, ** $P < 0.01$, *** $P < 0.001$. Summary data are expressed as median and interquartile range. (e) Small interfering RNA (siRNA)-mediated silencing of PTX3 does not affect viability in pancreatic stellate cells. PS1 cells were transfected with PTX3 siRNA or non-targeting control (NT) siRNA and viability was analysed by MTS assay. (f) Western blot of PTX3 expression in CM of PS1 transfected with PTX3 siRNA or non-targeting control (NT) siRNA from MTS assay cell cultures, and (g) densitometric quantification (n=3). Mann Whitney U test, *** $P < 0.001$. Summary data are expressed as mean with standard error mean.

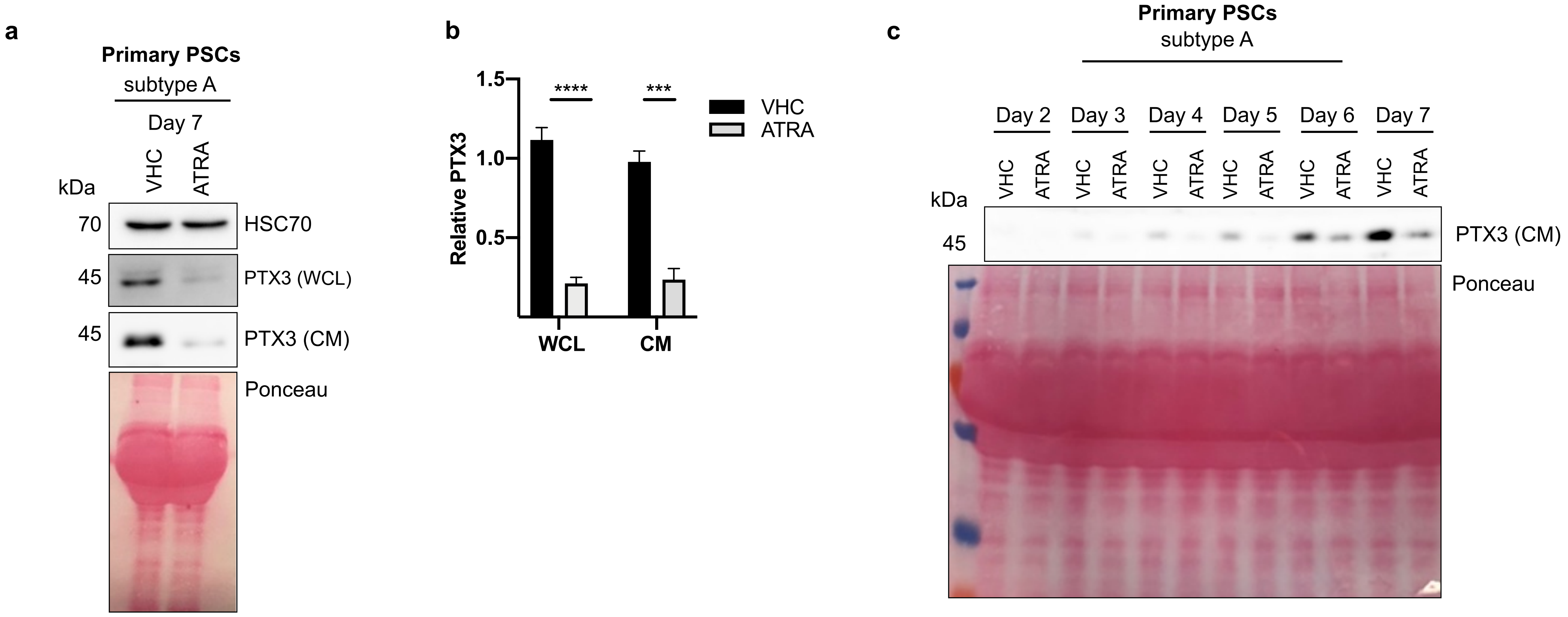

Supplementary Figure 7: PTX3 modulation after treatment with ATRA. ATRA treatment decreases PTX3 expression in primary stellate cells. (a) Western blot of PTX3 expression in whole cell lysates (WCL) and conditioned media (CM) of primary stellate cell subtype A after 7 days of ATRA or vehicle (VHC) treatment and (b) densitometric quantification (n=4). (c) Western blot of PTX3 expression conditioned media (CM) of primary stellate cell subtype A after ATRA or vehicle (VHC) treatment from Day 2 to Day 7. Mann Whitney U test,***P < 0.001, **** P < 0.0001. Summary data are expressed as mean with standard error mean.

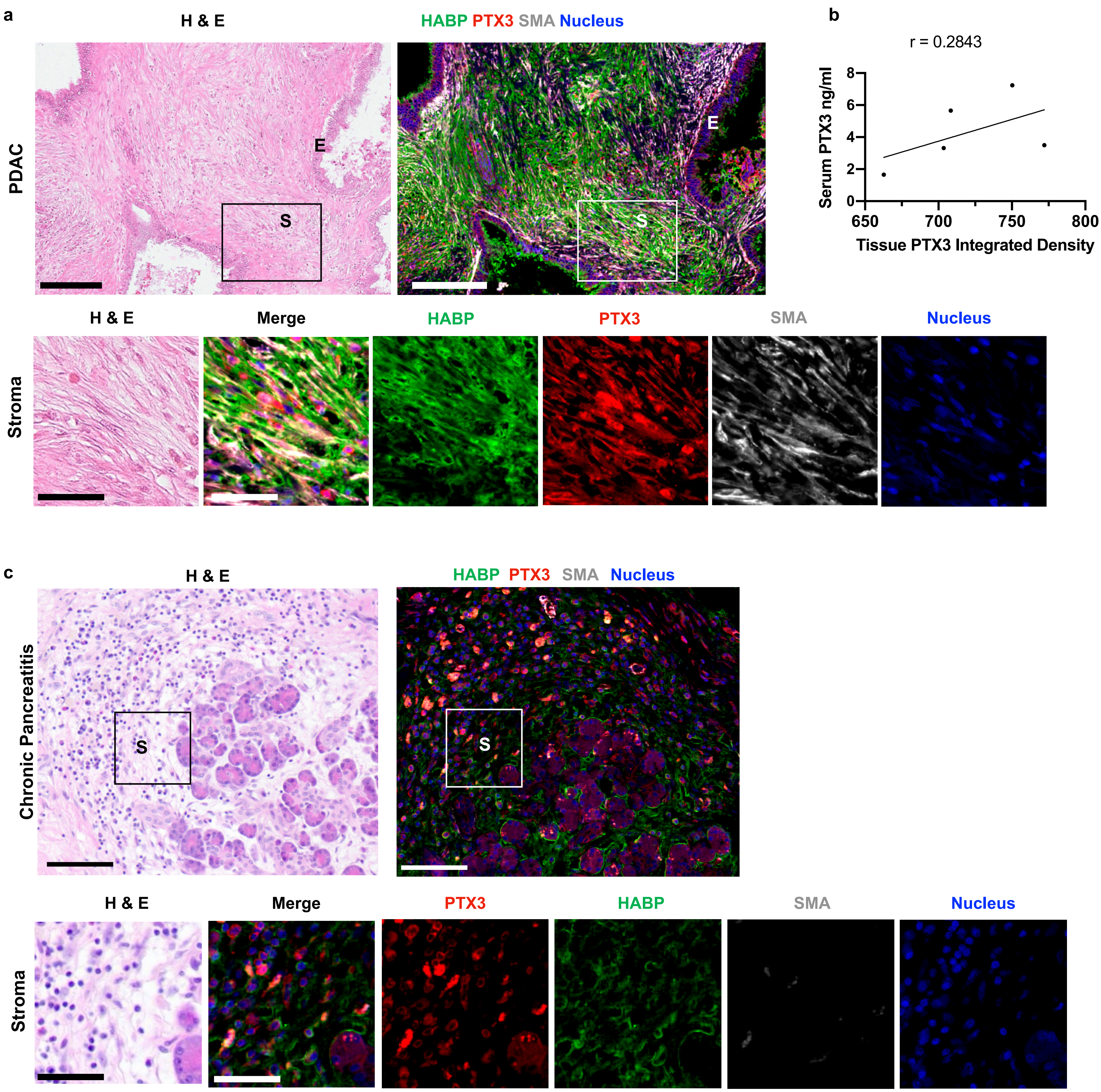

Supplementary Figure 8: HABP and PTX3 distribution in human PDAC and chronic pancreatitis. (a) Representative histological image (H&E) of human PDAC tissue and respective immunofluorescence staining, showing expression of HABP, PTX3 and α-SMA with (*Scale bar* = 250μm) with magnification of selected area (*Scale bar* = 50μm), E: Epithelial, S: stroma. Representative images also shown on supplementary videos 1, 2, and 3. (b) Correlation plot between serum PTX3 levels and tissue PTX3 expression in PDAC samples (n = 5) Pearson r = - 0.2843. (c) Representative histological image (H&E) of human pancreatitis tissue and respective iImmunofluorescence staining, showing expression of HABP, PTX3 and α-SMA with magnification of selected areas. *Scale bar* = 50μm. S: stroma. *Scale bar* = 100μm.

Supplementary Table1: Sensitivity, specificity and cut off values for PTX3, CA19-9, CEA, concurrent and sequential PTX3/ CA19-9.

Parameter	CEA	CA19-9	PTX3
**N**	133	141	162
**Cut off used***	2.06	37	4.35
**AUC (%)**	0.64	0.84	0.91
**Sensitivity (%)**	65	85	86
**Specificity (%)**	54	72	86
**Likelihood ratio**	1.42	3.13	6.05
**PPV (%)**	67.8	85.7	97.5

*** CEA in µg/mL, CA19-9 in IU/mL. PTX3 in ng/mL. AUC: area under the curve, PPV: positive predictive value.**

Supplementary Table 2: PTX3 small interfering RNA sequences.

ON-TARGETplus PTX3 siRNA - SMARTpool	Sequences
J-0177765-09	GUGAAUUUGGACAAGCAAA
J-0177765-10	CUGCAGUGUUGGCCGAGAA
J-0177765-11	GGUCAGGAGCACUCGGAAU
J-0177765-12	GGAUAGUGUUCUUAGCAAU

Supplementary Table 3: Clinical correlates with serum PTX3 levels.

	Spearman r	P value
**Albumin**	0.01	0.94
**Bilirubin**	-0.13	0.47
**WCC**	0.14	0.48
**CRP**	0.32	0.20
**Vitamin E**	-0.20	0.19
**Vitamin A**	-0.34	0.02

Supplementary Table 4: SCALOP patient characteristics for patients with and without biomarker data.

Relationship to whole trial population (N=114).

		Patients with Biomarker Data (n=85)		Patients without Biomarker Data (n=29)		Total (N=114)	
**Treatment**	Gemcitabine	29	34%	9	31%	38	33%
	Capecitabine	26	31%	10	34%	36	32%
	Missing	30	35%	10	34%	40	35%
**Sex**	Male	47	55%	16	55%	63	55%
	Female	38	45%	13	45%	51	45%
	Missing	0	0%	0	0%	0	0%
**Age**	<65	46	54%	14	48%	60	53%
	≥65	39	46%	15	52%	54	47%
	Missing	0	0%	0	0%	0	0%
**WHO Performance Status**	0	42	49%	13	45%	55	48%
	1-2	43	51%	16	55%	59	52%
	Missing	0	0%	0	0%	0	0%
**Median CA19-9 (U/mL),** IQR		379.0 (72.0, 978.0)		201.0 (88.5, 1463.0)		331.5 (76.8, 1051.8)	
**Median longest diameter of disease (cm),** IQR		4.0 (3.1, 5.0)		4.0 (3.0, 5.0)		4.0 (3.0, 5.0)	

Supplementary Table 5: SCALOP patient characteristics for patients by week 17 with respect to disease progression.

		Disease Progression at Week 17				*p-value*
		Yes (n=17)		No (n=63)		
**Sex**	Male	10	59%	35	56%	1.000
	Female	7	41%	28	44%	-
	Missing	0	0%	0	0%	-
**Age**	<65	10	59%	34	54%	0.789
	≥65	7	41%	29	46%	-
	Missing	0	0%	0	0%	-
**WHO Performance Status**	0	4	24%	13	56%	0.028
	1-2	13	76%	28	44%	-
	Missing	0	0%	0	0%	-
**CA19-9 (U/mL),** med IQR		442.0 (46.5, 1479.5)		331.5 (77.0, 889.8)		0.834
**Longest disease diameter (cm),** medIQR		4.4 (3.8, 5.9)		3.9 (3.0, 4.9)		0.095

Supplementary Table 6: Comparison of baseline PTX3 expression as a predictor biomarker for progression by week 17.

Parametric methods were used as the distribution of PTX3 was approximately normal after log transformation.

Log transformed	Progressed				Not Progressed				*p-value*
	**n**	**Mean**	**SD**	**95% CI**	**n**	**Mean**	**SD**	**95% CI**	
PTX3	17	1.27	1.17	(-1.03, 3.57)	63	1.340	1.12	(-0.85, 3.53)	0.830

Supplementary Table7: Patient characteristics for randomized patients with and without PTX3 data.

		Randomised Patients with Biomarker Data (n=64)		Randomised Patients without Biomarker Data (n=10)		Total (n=74)	
**Treatment**	Gemcitabine	33	52%	5	50%	38	51%
	Capecitabine	31	48%	5	50%	36	49%
	Missing	0	0%	0	0%	0	0%
**Sex**	Male	36	56%	5	50%	41	55%
	Female	28	44%	5	50%	33	45%
	Missing	0	0%	0	0%	0	0%
**Age**	<65	36	56%	2	20%	38	51%
	≥65	28	44%	8	80%	36	49%
	Missing	0	0%	0	0%	0	0%
**WHO Performance Status**	0	37	58%	3	30%	40	54%
	1	27	42%	7	70%	34	46%
	Missing	0	0%	0	0%	0	0%
**Median CA19-9 (U/mL),** IQR		235 (72, 822)		139 (82, 720)		212 (73, 815)	
**Median longest diameter of disease (cm),** IQR		3.8 (3.0, 4.9)		4.4 (3.3, 4.7)		3.9 (3.0, 4.9)	

Supplementary Table 8: Baseline, week 17, week 23 & week 39 PTX3 expression for randomised patients.

Univariate Cox proportional hazard models were fitted for PTX3 at each timepoint as continuous variables, which showed that there was no evidence to suggest that PTX3 was associated with overall survival and thus none were taken forward for further analyses.

Biomarkers	n	Mean	SD	95% CI	Median (IQR)	Hazard Ratio (95% CI)	*p-value*	*FDR*
PTX3 Baseline	55	6.76	7.32	(-7.59, 21.12)	4.96 (1.52, 8.76)	1.00 (0.97, 1.04)	0.871	0.992
PTX3 W17	49	3.95	3.79	(-3.48, 11.38)	2.77 (1.71, 4.69)	1.00 (0.91, 1.09)	0.992	0.992
PTX3 W23	40	4.86	5.68	(-6.28, 16.00)	3.17 (1.96, 5.14)	1.05 (0.98, 1.13)	0.136	0.546
PTX3 W39	22	4.75	5.08	(-5.19, 14.70)	2.69 (1.34, 6.97)	1.03 (0.96, 1.11)	0.439	0.878

Supplementary Table 9: Correlation of serum PTX3 with absolute neutrophil count (ANC).

Biomarkers	ANC	
	n	r – Pearson's correlation coefficient
PTX3 Baseline	55	0.3
PTX3 W17	47	0.1
PTX3 W23	37	0.2
PTX3 W39	21	0.1

The correlation coefficients are all less than or equal to 0.3, which indicates a low correlation between PTX3 and ANC at each timepoint.

Supplementary Video 1: HABP, PTX3, SMA distribution in human PDAC. (A) Representative immunofluorescence staining showing expression of HABP, PTX3 and α-SMA in human PDAC tissue with magnification of selected areas. (top) *Scale bar* = 250μm. (left, right) *Scale bar* = 50μm.

Supplementary Video 2: HABP, PTX3, E-cadherin distribution in human PDAC. (A) Representative immunofluorescence staining showing expression of HABP, PTX3 and E-cadherin in human PDAC tissue with magnification of selected areas. (top) *Scale bar* = 250μm. (left, right) *Scale bar* = 50μm.

Supplementary Video 3: HABP, PTX3, TSG-6 distribution in human PDAC. (A) Representative immunofluorescence staining showing expression of HABP, PTX3 and TSG-6 in human PDAC tissue with magnification of selected areas. (top) *Scale bar* = 250μm. (left, right) *Scale bar* = 50μm.
