# Supplementary material for: Pentraxin 3 is a stromally-derived biomarker for detection of pancreatic ductal adenocarcinoma
Source: NPJ Precis Oncol. 2021 Jun 29;5:61. doi: 10.1038/s41698-021-00192-1 (PMC8242009; doi:10.1038/s41698-021-00192-1)
Supplement: Supplementary file 2 — Reporting Summary [file 41698_2021_192_MOESM2_ESM.pdf]

nature research

Corresponding author(s): Hemant Kocher

Last updated by author(s): Apr 4, 2020

Reporting Summary

Nature Research wishes to improve the reproducibility of the work that we publish. This form provides structure for consistency and transparency in reporting. For further information on Nature Research policies, see Authors & Referees and the Editorial Policy Checklist.

Statistics

For all statistical analyses, confirm that the following items are present in the figure legend, table legend, main text, or Methods section.

n/a	Confirmed	
☐	☒	The exact sample size (*n*) for each experimental group/condition, given as a discrete number and unit of measurement
☐	☒	A statement on whether measurements were taken from distinct samples or whether the same sample was measured repeatedly
☐	☒	The statistical test(s) used AND whether they are one- or two-sided<br>*Only common tests should be described solely by name; describe more complex techniques in the Methods section.*
☐	☒	A description of all covariates tested
☐	☒	A description of any assumptions or corrections, such as tests of normality and adjustment for multiple comparisons
☐	☒	A full description of the statistical parameters including central tendency (e.g. means) or other basic estimates (e.g. regression coefficient) AND variation (e.g. standard deviation) or associated estimates of uncertainty (e.g. confidence intervals)
☒	☐	For null hypothesis testing, the test statistic (e.g. *F*, *t*, *r*) with confidence intervals, effect sizes, degrees of freedom and *P* value noted<br>*Give P values as exact values whenever suitable.*
☒	☐	For Bayesian analysis, information on the choice of priors and Markov chain Monte Carlo settings
☒	☐	For hierarchical and complex designs, identification of the appropriate level for tests and full reporting of outcomes
☒	☐	Estimates of effect sizes (e.g. Cohen's *d*, Pearson's *r*), indicating how they were calculated

Our web collection on statistics for biologists contains articles on many of the points above.

Software and code

Policy information about availability of computer code

Data collection	All clinical data were collected password protected Microsoft Excel spreadsheets,
Data analysis	All data were analysed using PRISM (GraphPad Inc) version 8.

For manuscripts utilizing custom algorithms or software that are central to the research but not yet described in published literature, software must be made available to editors/reviewers. We strongly encourage code deposition in a community repository (e.g. GitHub). See the Nature Research guidelines for submitting code & software for further information.

Data

Policy information about availability of data

All manuscripts must include a data availability statement. This statement should provide the following information, where applicable:

- Accession codes, unique identifiers, or web links for publicly available datasets
- A list of figures that have associated raw data
- A description of any restrictions on data availability

The data that support the findings of this study are available from the corresponding author upon reasonable request. There are no publicly available datasets for this work.

Field-specific reporting

Please select the one below that is the best fit for your research. If you are not sure, read the appropriate sections before making your selection.

☒ Life sciences ☐ Behavioural & social sciences ☐ Ecological, evolutionary & environmental sciences

Life sciences study design

All studies must disclose on these points even when the disclosure is negative.

Sample size	A pilot study assessing serum PTX3 levels in patients with PDAC along with age-/gender-matched controls (Figure 1A) and nomograms for diagnostic tests 10, determined a sample size of 260. This was based on an assumption of 50% prevalence (P~0.5) in our cohort (cancer versus other pancreatic diseases or normal controls), an anticipated accuracy (W~0.05) and a confidence interval of 5% (CI=0.05, z=1.96) with a sensitivity of 90% (SN=0.9), and specificity of 90% (SP=0.9) 13,14.
Data exclusions	No data were excluded. Where data is not available (reduced n), the correct 'n' is mentioned in each figure.
Replication	All Coefficient of variation are reported in respective sections.
Randomization	Not applicable.
Blinding	Prospectively collected samples obtained from the UK Medicine and Healthcare products Regulatory Agency approved SCALOP (ISRCTN 96169987) clinical trial 22 and STARPAC 23 were analyzed blindly and serum PTX3 assay data sent back to respective clinical trial units for clinical correlates.

Reporting for specific materials, systems and methods

We require information from authors about some types of materials, experimental systems and methods used in many studies. Here, indicate whether each material, system or method listed is relevant to your study. If you are not sure if a list item applies to your research, read the appropriate section before selecting a response.

Materials & experimental systems

n/a	Involved in the study	
☐	☒	Antibodies
☐	☒	Eukaryotic cell lines
☒	☐	Palaeontology
☒	☐	Animals and other organisms
☐	☒	Human research participants
☐	☒	Clinical data

Methods

n/a	Involved in the study	
☒	☐	ChIP-seq
☒	☐	Flow cytometry
☒	☐	MRI-based neuroimaging

Antibodies

Antibodies used	Western blotting: PTX3 (Cat. No HM2242, clone MNB4; Hycult Biotech), TSG-6 (Cat. No PA5-47253, ThermoFisher) or HSC70 (Cat. No sc-7298, Santa Cruz).<br>Immunoflourescence:
Validation	Described in methods. For staining of tissue sections: Organotypic sections, as previously described (Carapuca E et al, J Pathol 2016), were used for positive and negative staining controls. Controls were uniformly negative with appropriate isotype-specific immunoglobulin at matching dilutions.<br><br>For ELISA: PTX3 levels were quantified with a sandwich ELISA using in-house validated protocol based on a monoclonal antibody MNB4 (Latini, Circulation 2004) .

Eukaryotic cell lines

Policy information about cell lines

Cell line source(s)	ATCC
Authentication	STR profile: LGC Biosciences
Mycoplasma contamination	in house testing
Commonly misidentified lines<br>(See ICLAC register)	not applicable

Human research participants

Policy information about studies involving human research participants

Population characteristics	Described in respective clinical trials; STARPAC and SCALOP
Recruitment	Described in respective clinical trials; STARPAC and SCALOP
Ethics oversight	Described in respective clinical trials; STARPAC and SCALOP

Note that full information on the approval of the study protocol must also be provided in the manuscript.

Clinical data

Policy information about clinical studies

All manuscripts should comply with the ICMJE guidelines for publication of clinical research and a completed CONSORT checklist must be included with all submissions.

Clinical trial registration	ISCRTN 96169987 for SCALOP and NCT03307148 for STARPAC
Study protocol	Mukherjee, S., et al. Gemcitabine-based or capecitabine-based chemoradiotherapy for locally advanced pancreatic cancer (SCALOP): a multicentre, randomised, phase 2 trial. Lancet Oncol 14, 317-326 (2013).<br><br>Kocher, H.M., et al. Phase I clinical trial repurposing all-trans retinoic acid as a stromal targeting agent for pancreatic cancer. Nat Commun 11, 4841 (2020).
Data collection	Mukherjee, S., et al. Gemcitabine-based or capecitabine-based chemoradiotherapy for locally advanced pancreatic cancer (SCALOP): a multicentre, randomised, phase 2 trial. Lancet Oncol 14, 317-326 (2013).<br><br>Kocher, H.M., et al. Phase I clinical trial repurposing all-trans retinoic acid as a stromal targeting agent for pancreatic cancer. Nat Commun 11, 4841 (2020).
Outcomes	Mukherjee, S., et al. Gemcitabine-based or capecitabine-based chemoradiotherapy for locally advanced pancreatic cancer (SCALOP): a multicentre, randomised, phase 2 trial. Lancet Oncol 14, 317-326 (2013).<br><br>Kocher, H.M., et al. Phase I clinical trial repurposing all-trans retinoic acid as a stromal targeting agent for pancreatic cancer. Nat Commun 11, 4841 (2020).
